# Bi-frontal transcranial alternating current stimulation in the ripple range reduced overnight forgetting

**DOI:** 10.3389/fncel.2015.00374

**Published:** 2015-09-24

**Authors:** Géza Gergely Ambrus, Alberto Pisoni, Annika Primaßin, Zsolt Turi, Walter Paulus, Andrea Antal

**Affiliations:** ^1^Clinic for Clinical Neurophysiology, University Medical Center, Georg-August-University of GöttingenGöttingen, Germany; ^2^University Medical Center, Institute of Medical Psychology and Medical Sociology, Georg-August-University of GöttingenGöttingen, Germany; ^3^Institute of Psychology, Friedrich-Schiller-University of JenaJena, Germany; ^4^Department of Psychology, University of Milan BicoccaMilan, Italy

**Keywords:** transcranial alternating current stimulation, tACS, verbal associative learning, ripple oscillation, declarative memory

## Abstract

High frequency oscillations in the hippocampal structures recorded during sleep have been proved to be essential for long-term episodic memory consolidation in both animals and in humans. The aim of this study was to test if transcranial Alternating Current Stimulation (tACS) of the dorsolateral prefrontal cortex (DLPFC) in the hippocampal ripple range, applied bi-frontally during encoding, could modulate declarative memory performance, measured immediately after encoding, and after a night's sleep. An associative word-pair learning test was used. During an evening encoding phase, participants received 1 mA 140 Hz tACS or sham stimulation over both DLPFCs for 10 min while being presented twice with a list of word-pairs. Cued recall performance was investigated 10 min after training and the morning following the training session. Forgetting from evening to morning was observed in the sham condition, but not in the 140 Hz stimulation condition. 140 Hz tACS during encoding may have an effect on the consolidation of declarative material.

## Introduction

Memory formation is a dynamic process, which requires continuous encoding of new pieces of information and subsequent long-term consolidation (Frankland and Bontempi, 2005; Axmacher et al., 2008; Kuhl et al., 2010; O'Neill et al., 2010). The formation of a stable representation of the encoded material involves temporally-structured and precisely coordinated communication between the hippocampal structures and the neocortex (Sirota et al., 2003; Frankland and Bontempi, 2005; Logothetis et al., 2012). This dialogue is characterized by slow-frequency (0.5–4 Hz) and high-frequency oscillations (or so-called ripples; 100–200 Hz), although each oscillation is believed to serve different functions within the mnemonic process (Buzsáki, 1989; O'Neill et al., 2010).

Hippocampal ripple oscillations have been observed in both animals and humans and seem to have a critical role in the reiteration and consolidation of previously acquired information (Buzsáki, 1998; Staba et al., 2002). Evidence coming from animal studies and human intracranial recordings, indicate a temporal association between ripples generated in the hippocampus and sleep associated cortical oscillations in the neocortex occurring during sleep, and that this process might reflect the transfer of memory representations from the hippocampus to various neocortical regions including the frontal and temporal lobes (Axmacher et al., 2008). This combined activity outlines a pattern of synchronized up-regulations in several cortical foci, including mainly association cortices, parietal and early sensory cortices (but not the primary visual cortex). It is combined with a series of down-regulated oscillations in subcortical structures, like the thalamus, the basal ganglia, and the midbrain- brainstem (Logothetis et al., 2012). Results from animal studies suggest that the experimental alteration of these precisely orchestrated and temporally structured oscillations can lead to functionally relevant changes in the animal's behavior (Buzsáki, 1989; Girardeau et al., 2009; Jadhav et al., 2012; Varga et al., 2012).

Hypotheses derived from animal experiments with deep brain stimulation can now be directly tested on humans by the application of transcranial electric stimulation techniques mimicking intrinsic oscillatory activity (e.g., Polanía et al., 2012; Neuling et al., 2013; Helfrich et al., 2014; Strüber et al., 2014). Transcranial Alternating Current Stimulation (tACS), a specific subtype of Non-invasive Brain Stimulation methods (NIBS), is based on the application of low-intensity electrical currents oscillating sinusoidally at a predetermined frequency (Antal et al., 2008; for an overview see Paulus, 2011; Brignani et al., 2013). TACS-mediated physiological and behavioral changes seems to be frequency-dependent, thus, tACS could interact with the on-going brain activity through cortical oscillatory entrainment (Kirov et al., 2009; Pogosyan et al., 2009; Zaehle et al., 2010, 2011; Strüber et al., 2014), even if evidence in this direction is still controversial (Brignani et al., 2013).

Possible effects of transcranially applied oscillating currents on memory functions have been investigated on humans by using transcranial Slow Oscillation Stimulation (SO-tDCS; i.e., anodal transcranial direct current stimulation oscillating at 0.75 Hz in a trapezoid waveform-fashion, applied bi-frontally) in combination with on-line EEG recording during slow wave sleep (Marshall et al., 2006). Enhanced memory consolidation and increased learning performance was found when using slow-oscillating stimulation, which was applied during slow-wave sleep in the night (Marshall et al., 2004) or during an afternoon nap (Antonenko et al., 2013), which was also accompanied by the enhancement of the endogenous EEG slow oscillatory activity (0.5–1 Hz). A recent study, however, could not replicate these findings (Sahlem et al., 2015).

Ripple-range oscillations in the hippocampus have also been associated with declarative memory consolidation (Axmacher et al., 2008). The present study investigates the effects of tACS on memory performance, applied in the hippocampal ripple frequency range, specifically at 140 Hz, in awake participants during the encoding phase of a word-pair learning task. Our aim was not to target subcortical structures via tACS *directly*, since this technique is not able to focally target deep brain structures. Instead, we aimed to act on the prefrontal areas of both hemispheres, which are part of the network responsible for memory consolidation (Marshall et al., 2006; Murray and Ranganath, 2007; Takahashi et al., 2007). We hypothesized that stimulating the bilateral prefrontal cortices that are involved both in declarative memory encoding and sleep-dependent memory consolidation might result in a boosted memory performance when the stimulation is applied just before night sleep. Ten minutes of 140 Hz tACS at 1.0 mA has been previously shown to enhance cortical excitability in the motor system, as measured by Transcranial Magnetic Stimulation (TMS) elicited motor evoked potentials (MEPs, Moliadze et al., 2010). However, in a previous study, 140 Hz tACS had no modulatory impact on implicit learning performance in a serial reaction time task (Moliadze et al., 2012). Here, we aim to examine if tACS in the ripple range, applied during encoding and at a specific time-point (close before night sleep), is able to modulate declarative memory performance.

## Methods

### Participants

Eighteen participants (6 male, mean age: 24.61 ± 3.20 years) took part in the experiment. None of the participants reported previous history of neurological or psychological disorders, drug or alcohol abuse, had no metal implants and were not taking regular medication relevant to the study. Their visual acuities were normal or corrected to normal. Only native German speakers have been selected for the study. Informed consent has been acquired from all participants. The experiment had been conducted in accordance with the guidelines of the Declaration of Helsinki, and with the approval of the ethics committee of the University of Göttingen.

### Word-pair lists

The items of the verbal learning test have been obtained from the report by Marshall et al. (2006) (http://www.nature.com/nature/journal/v444/n7119/extref/nature05278-s2.pdf). Both word-pair lists contained 46 pairs of semantically related German nouns, in addition to four word-pairs at the beginning and at the end of each list acting as buffers against primacy and recency effects.

### Stimulus presentation

The stimulus presentation was identical to Marshall et al. (2006): the word-pairs were presented with a rate of one pair per 5 s with a 100 ms inter-stimulus interval (ISI). The order of stimulation conditions and the order of the word-pair lists have been randomly assigned to each participant in a counterbalanced manner.

### Transcranial alternating current stimulation

Stimulation was delivered by a CE-certificated, microprocessor-controlled multi-channel constant current stimulator (DC-STIMULATOR MC, neuroConn GmbH, Ilmenau, Germany). The current was transferred using two pairs of standard rubber electrodes placed in rectangular sponges soaked in isotonic sodium chloride solution. The electrodes have been placed over the left and right frontal regions (F3 and F4 according to the international 10–20 EEG placement system) and over the left and right mastoids. Frontal electrodes have been placed in sponges with an area of 25 cm^2^ (5 × 5 cm; current density: 0.04 mA/cm^2^); mastoidal sponge electrode coverings had an area of 35 cm^2^ (5 × 7 cm; 0.029 mA/cm^2^). Stimulation has been applied for 10 min with a frequency of 140 Hz (main experiment) with a peak-to-peak intensity of 1.0 mA. A ramp-up and -down phase of 15 s was used at the beginning and at the end of the stimulation. Sham stimulation was administered similarly, but the duration of the stimulation was only 30 s.

### Procedure

**Figure 1:**
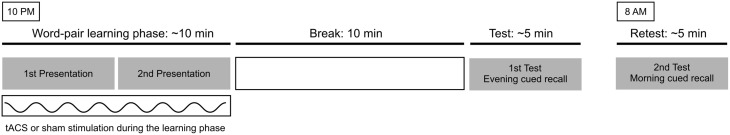
**The experimental procedure**. At 10 p.m., during 10 min of active or sham tACS stimulation the participants saw a word list two times. After an unfilled break of 10 min the subjects were tested on cued recall of the second word. Next morning at 8 a.m. they were tested again on the cued recall of the second word. The subjects participated in the study according to a repeated measures design.

Each of the experimental sessions consisted of a training and a retest part. The training part took place at 10 p.m. and consisted of a 10 min long learning and a 5 min long test phase. During the learning phase, one of the two lists of word-pairs was presented two times, during which active 140 Hz or sham tACS was applied. The cued-recall test phase followed the learning phase after an unfilled rest period of 10 min. In the test phase, the participant saw the first member of every word-pair (1 every 5 s, with 100 ms ISI) that was presented in the training phase, and had to recall the second, corresponding word. The participants were then asked to return home and sleep, and then come back the following morning at 08 a.m. for the retest session, in which the same word list had been presented for cued recall. Until the end of the second session they were asked to avoid drinking alcohol or caffeine. The order of word-pairs and cue-words had been randomized for every presentation. There was a minimum of 1-week break between the two stimulation sessions (mean ± SD: 12 days ± 7.92). (See Figure 1 for an overview of the experimental procedure).

### Statistical analyses

The analysis of the data was carried out using SPSS, version 22 (Armonk, NY: IBM Corp).

A repeated measures ANOVA was performed on the number of correctly recalled items with, as within subjects factors, Time (2 levels: evening and morning), and Condition (sham and active tACS). Differences between conditions have been investigated by means of *post-hoc* tests on estimated marginal means (Bonferroni correction applied). A second, separate, analysis was performed in the same way on subjects performing better than 60% at the evening sessions as in Marshall et al. (2006). Raw correct response data is given in Supplementary Table 1.

Values reported in the main text are means ± standard deviations. Statistical analyses were conducted using a significance level of *p* < 0.05.

## Results

**Figure 2:**
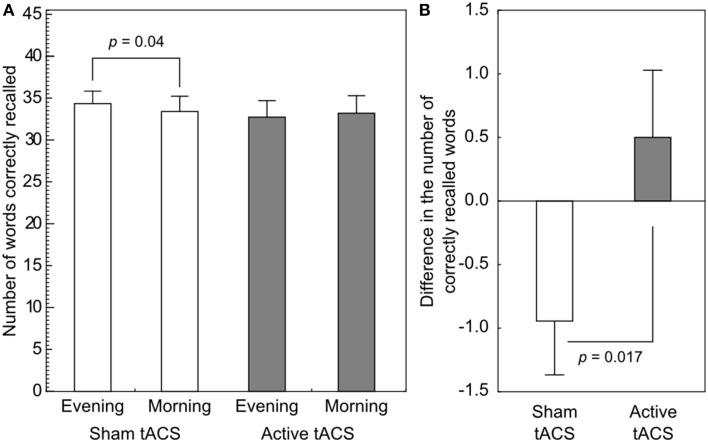
**Performance on the associative word-pair learning task**. **(A)** A paired *t*-test revealed a significant difference in performance in the sham stimulation condition, indicating forgetting. No such difference could be observed in the active stimulation condition. **(B)** The difference in the number of correctly recalled words from evening to morning was shown to be significantly different between the stimulation conditions. Error bars denote SEM.

Results are shown in Figure 2. In the sham stimulation condition participants were able to recall 74.76% (34.39 ± 6.09 words) of the items in the evening test phase, and 72.71% (33.44 ± 7.53 words) of the items in the retest session the following morning (difference: −2.05%, −0.94±1.80 words). In the active stimulation condition participants performed at 71.26% (32.78 ± 8.16 words) in the evening test phase and 72.22% (33.22 ± 8.72 words) in the morning retest phase (difference: 0.97%, 0.44 ± 2.84 words).

The ANOVA on the number of correctly recalled items showed that there was no main effect of Stimulation [*F*_(1, 17)_ = 0.338, *p* = 0.569] or change in performance from the evening to the next morning [*F*_(1, 17)_ = 0.326, *p* = 0.575] test sessions. The interaction between Time and Stimulation revealed a significant difference [*F*_(1, 17)_ = 7.004, *p* = 0.017, $\eta_{\text{p}}^{2}=0.29$, power = 0.95]. *Post-hoc* comparisons revealed no significant difference between the evening and the morning sessions (*p* = 0.457) in the 140 Hz condition, however, the performance decreased significantly in the sham condition dropping from 74.8 to 72.7% (*p* = 0.04). Importantly, no difference was found in the immediate recall sessions between the sham and the 140 Hz active stimulation conditions (*p* = 0.348).

To make our results comparable to those of Marshall et al. (2006), we have retrospectively removed from our sample the data of those participants who failed to reach ~60% performance (~27 words) in the evening session of either the active or the sham stimulation condition. After removing the data of these low-performing individuals from the dataset, 14 participants (4 male, age: 23.50 ± 2.07 years, word-list and stimulation session order still balanced) remained in the sample. The repeated measures ANOVA showed no main effect of Stimulation [*F*_(1, 13)_ = 0.0, *p* > 0.99, $\eta_{\text{p}}^{2}<0.0001$] or Time [*F*_(1, 13)_ = 0.02, *p* = 0.87, $\eta_{\text{p}}^{2}=0.002$], but the interaction between these factors was significant [*F*_(1, 13)_ = 12.0, *p* = 0.004, $\eta_{\text{p}}^{2}=12.0$]. Nevertheless, the *post-hoc* comparisons have revealed no statistically significant differences between within-condition time-points (140 Hz Evening: 35.50 ± 5.19—Morning: 36.35 ± 5.25, *p* = 0.145; Sham Evening: 36.28 ± 4.56—Morning: 35.57 ± 6.13, *p* = 0.146), or between time-point across conditions (Evening 140 Hz—Sham: *p* = 0.62; Morning 140 Hz—Sham: *p* = 0.60).

**Figure 3:**
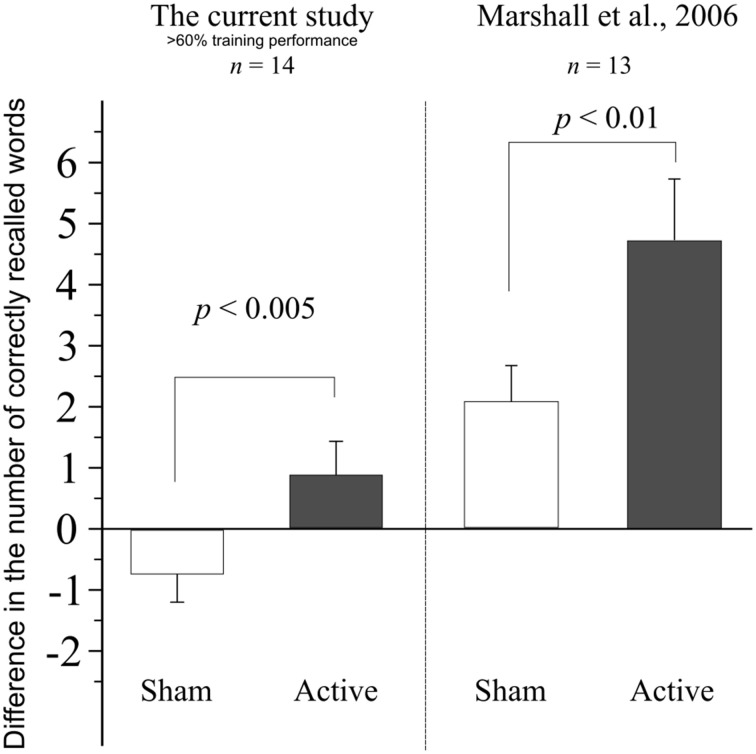
**Difference in performance between the evening and morning sessions, when data from subjects performing under 60% in the training session of either sham or active stimulation session are retrospectively removed**. Data from Marshall et al. (2006) shown for comparison. Error bars denote SEM.

Comparing the difference in accuracy in this high-performing sample, between the evening and morning sessions in the two stimulation conditions (sham: −0.71±1.73 words; active: 0.85 ± 2.07 words) using a paired *t*-test, we have found an enhancement of performance in the 140 Hz active condition that significantly differed from the reduction observed in the sham stimulation condition [*t*_(13)_ = 3.465, *p* = 0.004]. A comparison of our results with that of Marshall et al. (2006) is shown in Figure 3.

### Participants' blinding and self-reported measures of sleep quality

In order to assess whether the blinding was effectively maintained, participants were to provide a forced-choice post-experimental questionnaire after each stimulation condition (data available for the last 8 participants), where they could indicate the stimulation type (active or sham) they think they have received. The analysis revealed no significant difference between the two conditions in the number of active and sham answers as shown by a McNemar test (*p* = 1.0; active condition: 4 active/4 sham; sham condition: 3 active/5 sham).

In addition, the next morning participants completed a questionnaire about the self-reported measures of sleep quality (available for the last 6 participants). The questionnaire assessed the number of hours of sleep, the subjective quality of sleep, the time needed for falling asleep (in minutes) and the number of awakenings during sleep. The analysis revealed no significant difference between any of the measured values (Wilcoxon signed rank test, all *p*s > 0.109, Table 1).

**Table 1:** **The self-reported measures of sleep quality in the active and sham condition (*n* = 6)**.

	**Condition**	**Value**	**Wilcoxon-test**
Amount of sleep	Active	6.42 ± 0.58 (h)	*p* = 0.783
	Sham	6.50 ± 0.45 (h)	
Quality of sleep	Active	4.08 ± 0.92 (likert –scale)	*p* = 0.783
	Sham	3.83 ± 1.47 (likert –scale)	
Time to fall asleep	Active	9.50 ± 8.31 (min)	*p* = 0.109
	Sham	15.08 ± 10.96 (min)	
Number of awakenings	Active	1.08 ± 1.43 (times)	*p* = 0.197
	Sham	1.75 ± 1.75 (times)	

No significant difference was found as assessed by Wilcoxon-signed rank test.

## Discussion

In this study, we have demonstrated that 140 Hz tACS, applied bi-frontally over the DLPFC during encoding is able to reduce the overnight forgetting of learned material. Human NIBS studies have previously shown that the application of rTMS, or low-intensity electrical current stimulation such as tDCS, theta-tDCS, or tSOS might be effective tools for inducing changes in memory functions (Javadi and Walsh, 2012, for a review see Manenti et al., 2012). Here we suggest that frequencies other than low delta rhythms may improve declarative memory performance. Since ripple-range oscillations in the hippocampus have been temporally associated with cortical activations (Logothetis et al., 2012), tACS in the ripple frequency range seems to be an additional promising tool for inducing behavioral and, possibly, functionally relevant changes in the endogenous brain oscillation patterns. Indeed, a light flash triggered by hippocampal ripple activity, which disrupted ongoing neural activity and induced slower oscillatory activity resulted in a marked memory impairment in New Zealand White rabbits (Nokia et al., 2012).

Nevertheless, the effects of electrical stimulation seem to be brain state-dependent. Results from animal studies indicate that while theta oscillations are involved in the encoding processes in awake animals, slow-wave oscillations and ripples are believed to coordinate memory consolidation processes during sleep (Buzsáki, 1989; Girardeau et al., 2009). In humans, while tSOS (oscillating at 0.75 Hz) enhanced endogenous EEG activity and boosted sleep-related processes associated with episodic memory consolidation (Marshall et al., 2006; Antonenko et al., 2013), theta-tDCS (at a frequency of 5 Hz) caused decreased memory performance and a global decrease in slow oscillatory activity when the stimulation was applied during non-REM sleep (Marshall et al., 2011). When participants received tSOS in the consolidation period during wakefulness, the memory consolidation was not affected by the stimulation. On the other hand, when the participants received tSOS during memory encoding, the memory performance in immediate recall was enhanced (Kirov et al., 2009). It needs to be noted, however, that a recent study failed to replicate earlier results regarding the declarative memory-enhancing properties of oscillating bi-frontal anodal tDCS during slow wave sleep (Sahlem et al., 2015).

Our participants received tACS during the encoding phase while being awake, although close before the night's sleep. Therefore, it is difficult to determine if the effect of the stimulation was due to the aftereffects of the ripple-tACS on the following sleep consolidation period or if it was an on-line effect acting on the encoding process. As no difference has been observed between the immediate recall sessions in the active and sham stimulation conditions, it seems to be more plausible that the effect of stimulation, as small as it may be, was due to the effect exerted on the consolidation phase of the memory formation. It has been proven that the excitability changes induced by 140 Hz tACS over the motor cortex were still observable 1 h after the end of the stimulation (Moliadze et al., 2010). Even if it was not possible in our experimental setting to collect further data on the time-course of the stimulation aftereffects in our experimental setting, and we do not do we have knowledge of any other research investigating this specific issue outside of the motor domain, it is possible that these effects were still present during our subjects' sleep state. Nevertheless, it could be possible that enhancement of memory consolidation via ripple-range tACS would be more effective if participants received the stimulation during the consolidation period while in non-REM sleep rather than when being awake.

The aftereffects reported in the previously mentioned studies differ from our current results in the amount of the observed enhancement in performance. The reason for this difference might be due to several factors. First, stimulation was delivered before the sleep consolidation period, while Marshall et al. (2006) study administered it during sleep. Second the number of the presentation of the word pairs was different: while in Marshall et al. (2006) study stimuli were presented until subject reached an accuracy at least of 60%, we decided instead to present the word-pairs twice to fill the stimulation time (10 min). Since delivering the stimulation during the encoding phase was critical for the aims of our study, a fixed time for the presentation procedure was needed, preventing us from training our participants until a performance threshold was reached. Moreover, while Marshall and colleagues administered SO-tDCS, we decided to test the effects of ripple frequency stimulation, which has been associated to memory consolidation processes, using tACS. In addition, we used a lower current density in the current study (0.04 mA/cm^2^) compared to previous ones (0.517 mA/cm^2^) (Marshall et al., 2006, 2011; Kirov et al., 2009) and the stimulation waveform also differed (tSOS or theta-tDCS vs. tACS).

Our results might speculatively be explained by the following two mechanisms: as the study by Moliadze et al. (2012) demonstrated, 140 Hz stimulation resulted in an increase in an MEP size, possibly due to a reduction of the short intracortical inhibition (SICI), which was robust and persisted long after the end of the stimulation. Thus, a reduced inhibition of the stimulated areas might have resulted in a more efficient exchange between the subcortical structures leading to an enhanced consolidation of the encoded memories overnight. A second possible mechanism might be an increased synchronization between the hippocampus and cortical regions in the ripple frequency range, which might have enhanced memory consolidation. Indeed, as Logothetis et al. (2012) described in macaques, ripple-range synchronization can be observed in resting states and sleep during memory consolidation, and these oscillations are highly synchronized between hippocampus and cortical regions, and de-synchronized within subcortical areas. A sustained cortical entrainment (see Helfrich et al., 2014) of the prefrontal cortical regions might thus have boosted this mechanism, thereby enhancing memory consolidation.

The limitations of the present study are the following. (1) From this study alone it cannot be ascertained, whether the reduction in the amount of forgotten material associated with tACS was due to the direct modulation of the DLPFC itself (Javadi and Walsh, 2012), or the stimulation interacting with the hippocampus via proxy effects (Lang et al., 2005). The processes involved in declarative memory functions, indeed, are not isolated to a specific brain region, but instead, they are associated to highly interconnected neural circuits, involving even brain regions that are remote from one another (Cabeza and Nyberg, 2000; Bai et al., 2009; Henke, 2010). Although the direct effects of tACS might be too superficial to reach structures located as deep as the hippocampus, stimulation might have a modulating effect via the DLPFC, a key area in the hippocampal-neocortical interaction, through the prefrontal-limbic pathways. However, a performance-altering effect could have been achieved by the direct modulation of the excitability of the DLPFC, as it was demonstrated by NIBS studies, including repetitive TMS (rTMS, Sandrini et al., 2003) or transcranial direct current stimulation (tDCS, Javadi and Walsh, 2012). (2) Although tACS is considered to be a potential tool for entraining cortical oscillations, currently we have no direct evidence indicating that the physiological mechanisms observed at the behavioral level are due to cortical oscillation entrainment or to tACS-mediated cortical excitability changes (Moliadze et al., 2010; Brignani et al., 2013; Vossen et al., 2014). (3) Finally, there is evidence for hippocampal ripples to lie between 80 and 120 Hz in human subjects, (Axmacher et al., 2008) even if some studies reports frequencies up to 200 Hz in animals (Grenier et al., 2001; Sullivan et al., 2011) and to 140 Hz in humans (Staba et al., 2002; Clemens et al., 2007). tACS at a frequency of 140 Hz may be then too fast to modulate neural activity associated with enodogenous hippocampal ripples.

In summary, our results show that tACS in the ripple range is a promising technique to interact with memory processes. Investigating their effects on larger samples and with different paradigms is warranted in the future. A wider set of frequencies should also be tested to better refine the most effective band in modulating behavioral performance.

### Conflict of interest statement

The authors declare that the research was conducted in the absence of any commercial or financial relationships that could be construed as a potential conflict of interest.
